# Label-free Electrochemical Impedance Spectroscopy Aptasensor for Ultrasensitive Detection of Lung Cancer Biomarker Carcinoembryonic Antigen

**DOI:** 10.3389/fchem.2021.721008

**Published:** 2021-07-19

**Authors:** Yawei Wang, Lei Chen, Tiantian Xuan, Jian Wang, Xiuwen Wang

**Affiliations:** ^1^ Department of Medical Oncology, Qilu Hospital, Shandong University, Jinan, China; ^2^ Shandong Academy of Pharmaceutical Sciences, Jinan, China

**Keywords:** screen-printed carbon electrode, aptamer, carcinoembryonic antigen, EIS aptamer sensor, graphene nano-sheet

## Abstract

In this work, an integrated electrode system consisting of a graphene working electrode, a carbon counter electrode and an Ag/AgCl reference electrode was fabricated on an FR-4 glass fiber plate by a polyethylene self-adhesive mask stencil method combined with a manual screen printing technique. The integrated graphene electrode was used as the base electrode, and AuNPs were deposited on the working electrode surface by cyclic voltammetry. Then, the carcinoembryonic antigen aptamer was immobilized using the sulfhydryl self-assembly technique. The sensor uses [Fe(CN)_6_]^3−/4−^ as a redox probe for label free detection of carcinoembryonic antigen based on the impedance change caused by the difference in electron transfer rate before and after the binding of carcinoembryonic antigen aptamer and the target carcinoembryonic antigen. The results showed a good linear relationship when the CEA concentration is in the range of 0.2–15.0 ng/ml. The detection limit was calculated to be 0.085 ng/ml (S/N = 3).

## Introduction

In the early 1990s, scientists used *in vitro* screening techniques to isolate RNA and DNA molecules that specifically bind proteins, and these screened single-stranded oligonucleotides were called aptamers (Macugen Diabetic Retinopathy Study Group, 2005; Ostroff et al., 2010). Oligonucleotides are short single-stranded nucleic acid molecules that bind selectively and with high affinity to proteins or other target molecules. Aptamers have many characteristics that antibodies do not have and are often used as recognition elements for aptamer sensors (Pavlov et al., 2004; Zayats et al., 2006).

Aptamers are easy to synthesize and easy to modify compared to other specific recognition elements. In immunoassays, antibodies need to be obtained from animals and live cells. In contrast, nucleic acid aptamers are usually screened *in vitro* using the SELEX technique (Sampson, 2003; Darmostuk et al., 2015). In general, modification of antibodies leads to their inactivation. In contrast, modification of the aptamer does not affect either the activity or the binding of the aptamer to the target molecule (Zhang et al., 2018; Farzadfard et al., 2020). Unlike antibodies, which usually bind only to their corresponding antigens, aptamers can recognize different targets such as proteins Liu et al. (2020), peptides He et al. (2021), amino acids Idili et al. (2019), antibiotics Lin et al. (2018), small molecules Nakatsuka et al. (2018), viruses Chen et al. (2020), and even metal ions (Zhang Y. et al., 2020). The affinity of the aptamer for the target is far stronger than the binding force between the antibody and the antigen. In addition, aptamers are more stable than antibodies, which can be stored for longer periods of time at room temperature. Aptamers can regain their activity under appropriate conditions after denaturation (Belleperche and DeRosa, 2018). This feature of aptamers allows the lifespan of aptamer sensors to be extended.

Electrochemical aptasensor integrate the disciplines of biology, chemistry, physics, electronics and medicine (Karimi-Maleh et al., 2020; Kir, 2020; Ozbek et al., 2021). These disciplines support and permeate each other, which has led to the gradual development of electrochemical aptasensor (Zheng et al., 2018; Fu et al., 2019a; Zhang M. et al., 2020; Kizilgeci et al., 2020; Özkan, 2020; Karimi-Maleh et al., 2021b). Electrochemical aptamer sensor has the following advantages: fast analysis, simple operation method, good selectivity and high sensitivity. It is an analytical detection device constructed by combining an aptamer as a molecular recognition substance with electrochemical sensing (Fu et al., 2018, 2019b; Xu et al., 2020; Zheng et al., 2020). Therefore, electrochemical aptamer sensor has gradually become a research hotspot.

Currently, cancer remains the most feared disease in the world. In the early twenty-first century, prostate, lung, breast and colon cancers topped the list of deaths in the United States and Canada. In most developing countries, cancer was identified as the second leading cause of death. This led to the use of tumor markers Li et al. (2020a), Duan et al. (2021), Li et al. (2021), Wang et al. (2021), which are chemical-based substances that reflect the presence of tumors. They are not found in normal adult tissues but only in embryonic tissues Karimi-Maleh et al. (2021c), Karaman (2021), Karaman et al. (2021), or are present in tumor tissues at levels that greatly exceed those found in normal tissues (Li et al., 2020b; Vajhadin et al., 2020). Their presence or quantitative changes can reveal the nature of tumors, lend to the understanding of tumor histogenesis, cell differentiation and cell function, which can provide assistance in tumor diagnosis, classification and treatment guidance. Early diagnosis of cancer is crucial to successfully save patients’ lives. Therefore, sensitive and specific methods are needed to detect them. Disease detection is achieved by measuring the levels of biomarkers in blood, urine, and other body fluids. Carcinoembryonic antigen (CEA), an acidic glycoprotein with a relative molecular mass of 180 kDa, is of great importance for the development and monitoring of lung cancer (Yang et al., 2018; Song et al., 2020). Usually, the CEA content in biological samples is very low, and the threshold value of CEA in human serum is 5.0 ng/ml. When the CEA content in serum is greater than 5.0 ng/ml, it may be a precursor of lung cancer (Gu et al., 2018; Jozghorbani et al., 2021). Therefore, the detection of CEA is particularly important. So far, fluorescence analysis Qiu et al. (2017), radioimmunoassay Abu-Bakr El-Bayoumy et al. (2018), enzyme-linked immunoassay Wu et al. (2021), electrochemiluminescence Yang et al. (2020) and other methods have been used for CEA detection. Among these methods, electrochemical methods have attracted the interest of scientists due to the advantages of low cost and easy portability.

Graphene, a two-dimensional carbon material with high electron density, dielectric properties and catalytic effects, which make it widely used in biosensors (Mohanraj et al., 2020; Özcan et al., 2020). Most electrochemical aptamer sensors require labeling of the aptamer, which is a complicated process for labeling during experiments and may affect the specific binding of the aptamer to the target (Yang et al., 2017; Alavi-Tabari et al., 2018; Butmee et al., 2020; Naderi Asrami et al., 2020; Karimi-Maleh et al., 2021a). In recent years, label free aptamer sensors have attracted the interest of scientists because of their label-free, simple operation, fast detection speed and low cost (Rizwan et al., 2018). The main detection techniques used for label free aptamer sensors are electrochemical impedance spectroscopy (EIS) and square wave voltammetry coulometry. EIS has been widely developed and applied in the field of analytical chemistry for its high sensitivity (Singh et al., 2021).

In this work, an integrated thick film graphene electrode system consisting of a graphene working electrode, a large area carbon counter electrode and an Ag/AgCl reference electrode was fabricated by a polyethylene self-adhesive mask template method combined with screen printing technique. The integrated graphene electrode was used as the base electrode, and Au nanoparticle was deposited on the surface of the graphene working electrode by cyclic voltammetry. The CEA aptamer was immobilized by the sulfhydryl self-assembly technique, and [Fe(CN)_6_]^3−/4−^ was used as the probe. The EIS electrochemical aptamer sensor for label free detection of CEA was constructed based on the change of mass transfer resistance at the electrode before and after the binding of [Fe(CN)_6_]^3−/4−^ to the CEA.

## Materials and Methods

### Materials

All reagents were analytical grade and used without further purification. Carcinoembryonic antigen aptamer (CEA-aptamer) was purchased from Bioengineering Co.,Ltd. The sequence of the thiol-labeled CEA aptamer is: 5′-SH-ATACCAGCTTATTCAATT-3'. The carcinoembryonic antigen (CEA) was purchased from Shanghai Leadwave Biotechnology Co. 6-Methoxyl-1-hexanol (MCH) purchased from Sigma. Chloroauric acid (HAuCl_4_H_2_O), potassium ferricyanide (K_4_ [Fe(CN)_6_]·3H_2_O, potassium ferricyanide (K_3_ [Fe(CN)_6_]), dipotassium hydrogen phosphate (K_2_HPO_4_), potassium dihydrogen phosphate (KH_2_PO_4_) were purchased from Sinopharm Group Chemical Reagent Beijing Co.,Ltd. Graphene ink (Sheet diameter: 1–5 μm, Content: 5.0wt%, Solvent: NMP) was purchased from Nanjing XFNANO Materials Tech Co.,Ltd. Conductive silver adhesive purchased from Shanghai Baoyin Electronic Materials Co.,Ltd. FR-4 glass fiber board was purchased from Xi’an Xidian Electric Material Co.,Ltd. 5 mM K_3_ [Fe(CN)_6_]−5 mM K_4_ [Fe(CN)_6_]−0.1 M PBS (pH 7.0)−1.0 M KC1 was used as the impedance detection solution.

### Integrated Graphene Electrode Fabrication

Take the appropriate amount of conductive graphene ink and add it to the Petri dish, scrape it well with a scraper to get a uniform sticky conductive paste. The polyethylene self-adhesive presenter board is pasted onto a cleanly treated FR-4 glass fiberboard substrate (the fiberboard was washed with ethanol and distilled water and dried at room temperature). An integrated three-electrode system containing a graphene working electrode, a carbon counter electrode and a carbon reference electrode was obtained by screen printing technique. Then 1 layer of silver paste was evenly coated at the reference electrode and the prepared integrated electrode was dried in an oven at 70°C. An appropriate amount of 0.1 M FeCl_3_ solution was dropped on the silver surface, and the silver was oxidized to FeCl_3_. After 1 h, the FeCl_3_ solution was removed by rinsing with distilled water and air-dried to obtain the Ag/AgCl reference electrode. Finally, all areas except the working electrode, counter electrode, reference electrode and wire connection points were insulated with insulating tape.

### Preparation of Aptamer Sensors

**FIGURE 1:**
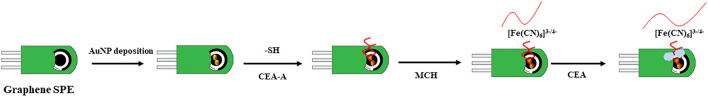
Preparation process of the aptamer sensor and the principle of detecting CEA.

The preparation process of the aptamer sensor and the principle of detecting CEA was shown in Figure 1. Firstly, the graphene SPE need to be activated. The SPE electrode was placed in 0.1 M PBS for CV scan. The scanning potential interval is between −0.5 and 1.5 V until a stable electrochemical signal was obtained. The treated SPE was placed in l mM HAuCl_4_ solution and electrodeposited by CV. CV scan was performed at a potential of −1.5–1 V to obtain Au nanoparticle modified SPE (Au/SPCE). The Au/SPE was washed with water and dried at room temperature, then 5 μL of l μM CEA-aptamer solution was pipetted onto the Au/SPE surface and incubated for 15 h. The working electrode was then washed with PBS buffer solution to remove the unbound aptamer to obtain CEA-A/Au/SPE. To prevent non-specific adsorption on the electrode surface, 5 μL of 20.0 nM MCH solution was coated to the electrode surface for 10 min to close the blank sites on the electrode surface. Then, the electrode surface was thoroughly washed with PBS buffer solution to remove the excess MCH solution. The aptamer sensor M/CEA-A/Au/SPE was obtained.

### Electrochemical Detection

C/M/CEA-A/Au/SPE was obtained by applying 10 μL of CEA solution coated on the surface of the assembled aptamer sensor and incubating it at 37°C for 1 h. The aptamer sensor was then washed with 0.01 M [Fe(CN)_6_] pH 7.4 PBS solution to remove the CEA adsorbed on the electrode surface. The aptamer sensor was placed in 15 ml of 5 mM [Fe(CN)_6_]^3−/4^–0.1 M PBS (pH 7.0)–0.1 M KC1 detection solution. The EIS was used for the detection. The frequency range and amplitude was 100 KHz∼0.1 Hz and 5.0 mV, respectively. The impedance theoretical value was fitted with the Randles equivalent circuit to analyze the detection signal for quantitative detection of CEA.

## Results and Discussion

**FIGURE 2:**
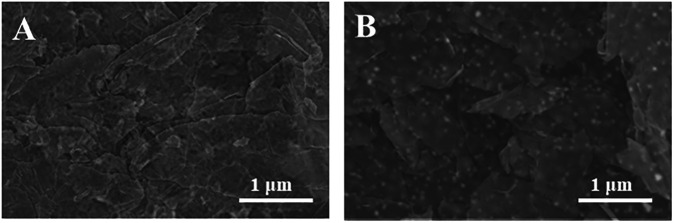
SEM images of SPE and Au/SPE.

The surface morphology of SPE and Au/SPE was characterized using scanning electron microscopy, and the results are shown in Figure 2. Figure 2A shows the SPE surface is relatively flat with a flaky distribution, which is consistent with the surface morphology of graphene (Kong et al., 2014). When AuNPs were electrodeposited on the graphene electrode surface, a large number of uniform particles appeared on the electrode surface (Figure 2B), indicating that AuNPs were successfully deposited on the SPE surface.

**FIGURE 3:**
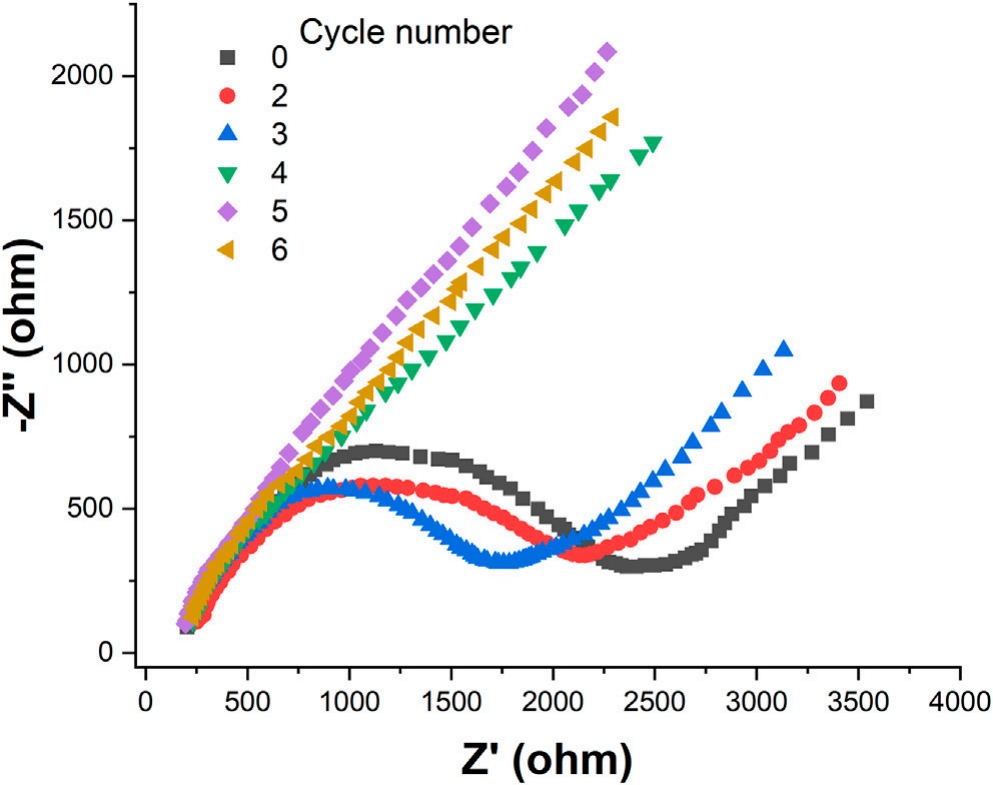
EIS of SPE and Au/SPE with different CV cycles recorded in 5 mM [Fe(CN)_6_]^3−/4−^.

Figure 3 shows the EIS of the SPE and Au/SPE with different CV cycles. It can be seen that the electron transfer resistance of [Fe(CN)_6_]^3−/4−^ on the SPE surface is relatively large. After the electrodeposition of AuNPs on the SPE surface, the electron transfer resistance of Au/SPE surface gradually decreases when the number of deposited cycle increases from 2 to 5, which indicates that the diffusion of [Fe(CN)_6_]^3−/4−^ in Au/SPE was accelerated. This is because AuNPs has high electron density and excellent dielectric properties, which promote electron transfer and increase the reversibility of redox substances on the electrode surface (Chan et al., 2016). When the number of deposition circles continues to increase, the electron transfer impedance of [Fe(CN)_6_]^3−/4−^ on the Au/SPE surface almost no longer changes, indicating that the amount of AuNPs deposited on the SPE surface almost reaches saturation.

**FIGURE 4:**
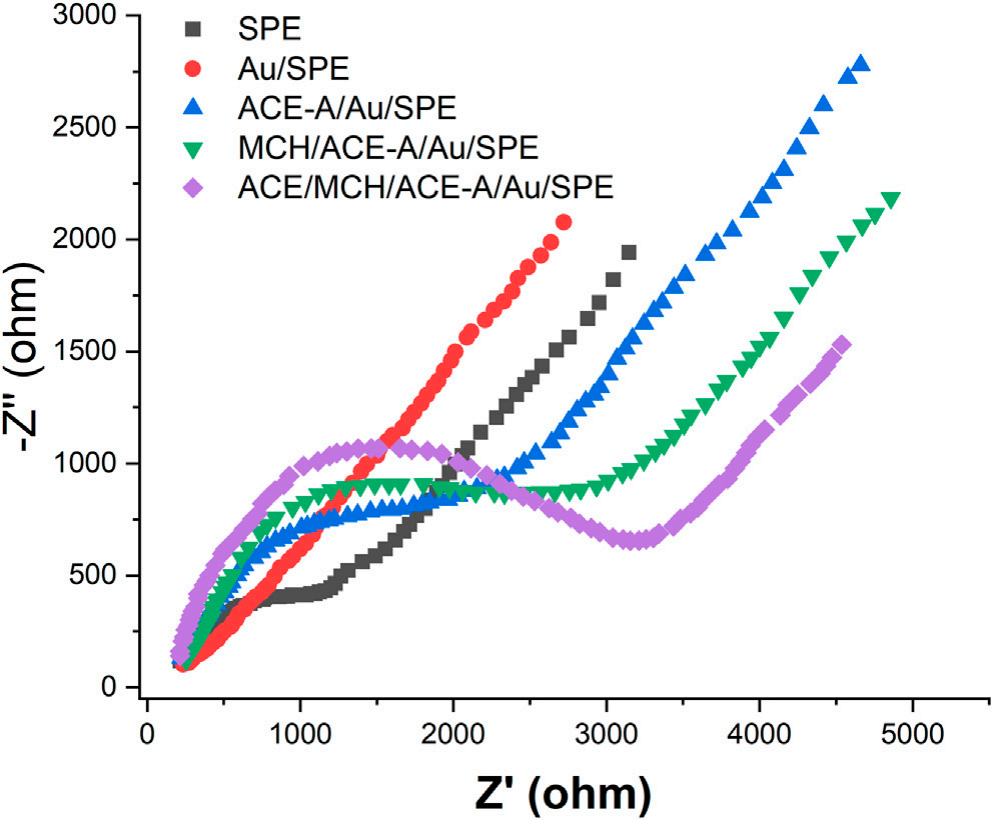
EIS plots of aptamer senor during the fabrication recorded in 5 mM [Fe(CN)_6_]^3−/4−^.

We then characterized the assembly process of the aptamer sensor using EIS, as shown in Figure 4 [Fe(CN)_6_]^3−/4−^ has a high electron transfer resistance at the SPE surface. When AuNPs were electrodeposited on the SPE surface, the electron transfer resistance decreased. This is because the AuNPs has excellent electrochemical properties, which accelerates the electron transfer rate of [Fe(CN)_6_]^3−/4−^ on the electrode surface. When CEA-A was immobilized on the electrode surface by the sulfhydryl self-assembly technique, the electron transfer of [Fe(CN)_6_]^3−/4−^ at the electrode surface was hindered. This is due to the fact that the aptamer is a negatively charged phosphate backbone, and [Fe(CN)_6_]^3−/4−^ mutually repel each other and hinder electron transfer (Yan et al., 2014). The impedance value further increases after using the sealer MCH to close the blank sites on the electrode surface that are not occupied by the aptamer. When l ng/mL CEA was incubated on the electrode, the impedance value continued to increase because the binding of the target CEA to the aptamer increased the spatial site resistance on the electrode surface and slowed down the electron transfer rate (Huang et al., 2012).

**FIGURE 5:**
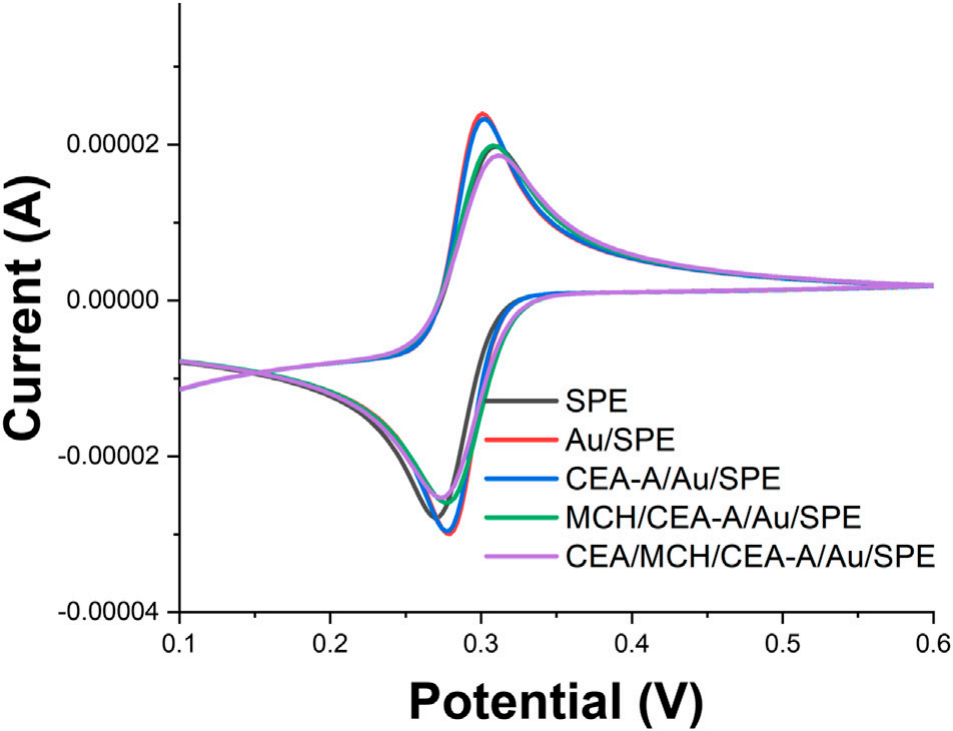
CV profiles of aptamer senor during the fabrication recorded in 5 mM [Fe(CN)_6_]^3−/4−^.

Also, we have characterized the whole assembly process using CV (Figure 5). It can be seen from Figure 5 that [Fe(CN)_6_]^3−/4−^ has good reversibility on the SPE. When AuNPs were electrodeposited on the SPE surface, the reversibility of [Fe(CN)_6_]^3−/4−^ on Au/SPE was further enhanced and the peak potential difference was significantly reduced. When the CEA-A with sulfhydryl groups self-assembled on the Au/SPE surface through Au-S bonds, the aptamer carrying a negatively charged phosphate backbone would repel the negatively charged [Fe(CN)_6_]^3−/4−^ and affect the electron transfer on the electrode surface, making the peak current smaller. Closure of the blank sites on the electrode surface not occupied by the aptamer with MCH formed a dense film on the electrode surface, which made the peak current further smaller. When 1 ng/ml of CEA was added, the electron transfer of [Fe(CN)_6_]^3−/4−^ was hindered, and the peak potential difference increased and the peak current decreased. After the addition of 10 ng/ml of CEA, the peak current continued to decrease.

**FIGURE 6:**
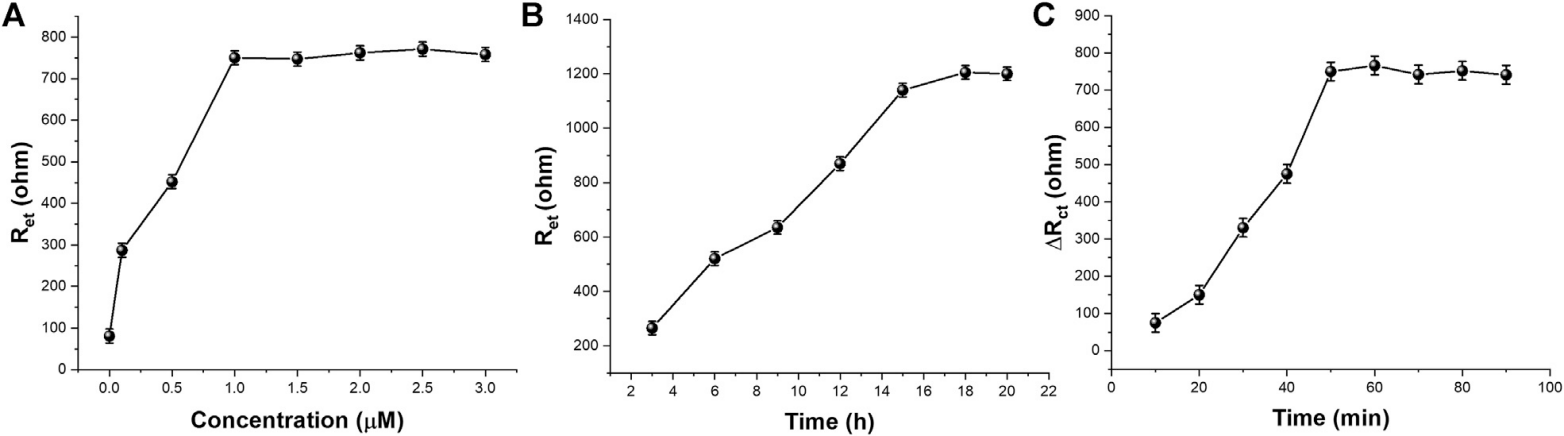
The effect of **(A)** CEA-A concentration **(B)** immobilization time and **(C)** incubation time on the aptasensor performance (*n* = 3).

The concentration of CEA-A immobilized on the surface of the electrode is an important factor affecting the performance of the sensor, so the CEA-A concentration was optimized. The relationship between the aptamer concentration of carcinoembryonic antigen and the electron transfer resistance (R_et_) is shown in Figure 6A. When the aptamer concentration increased from 0.1 to 1 μM, the Ret of [Fe(CN)_6_]^3−/4−^ on the sensor surface also increased gradually. When the aptamer concentration continued to increase to 30 μM, the impedance value reached a plateau and almost stopped changing. This indicates that the amount of CEA-A immobilized on the sensor surface has reached saturation. Therefore, the concentration of carcinoembryonic antigen aptamer was chosen to be 1 μM in subsequent experiments.

The immobilization time of the aptamer is an important factor that affects the performance of the aptamer sensor, therefore, the immobilization time of the aptamer was optimized. Figure 6B shows the relationship between the change of [Fe(CN)_6_]^3−/4−^ in the sensor surface heart after different times of immobilization of the CEA aptamer on the electrode surface for 1 μM. As can be seen from the figure, the R_et_ of the sensor gradually increases when the immobilization time of the CEA aptamer increases from 3 to 15 h. When the immobilization time increases from 15 to 21 h, the impedance value reaches a plateau and basically stops changing, indicating that the amount of aptamer immobilization has basically reached the saturation state. Therefore, the selected aptamer immobilization time was 15 h.

The binding time between the aptamer sensor and the target CEA is also an important factor affecting the performance of the sensor, so the binding time between the sensor and CEA was investigated in this experiment. The aptamer sensor was incubated with 10 μL of 0.5 ng/ml CEA at 37°C for different times, and the experimental results were shown in Figure 6C. It can be seen that when the incubation time was varied between 10–60 min, a significant increase in the electron transfer resistance occurred with the increase of time. When the incubation time was between 60–70 min, there was a small decrease in R_ct_. When incubation time excess 70 min, R_ct_ tended to be stable. This indicates that 70 min is a more reasonable time for specific binding of the aptamer to the target.

**FIGURE 7:**
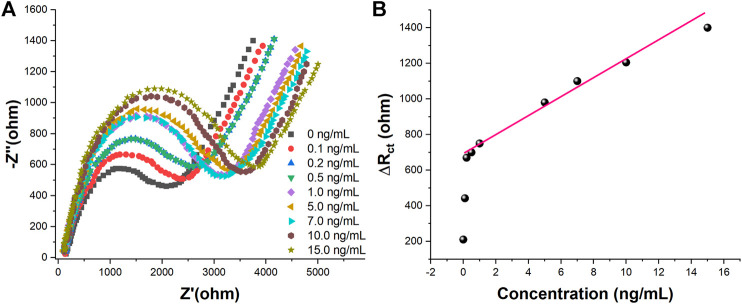
(A) EIS in 5 mM [Fe(CN)_6_]^3−/4–^0.1 M PBS (pH 7.0)−1 M KCl of the proposed aptasensor towards 0, 0.1, 0.2, 0.5, 1.0, 5.0, 7.0, 10.0, and 15.0 ng/ml of CEA (B)Plots of R_ct_ vs. CEA concentration.

Under the optimized experimental conditions, different concentrations of CEA were measured in 5.0 mM [Fe(CN)_6_]^3−/4–^0.1 M PBS (pH 7.0)−1 M KCl using the proposed aptamer sensor. The experimental results were shown in Figure 7. Figure 7A shows the EIS plots of the aptamer sensor combined with different concentrations of CEA. It can be seen from Figure 7A that the impedance value of the aptamer sensor in the blank solution is the smallest, and the impedance value increases gradually when different concentrations of the target CEA were added. This is because CEA binds to the aptamer immobilized on the aptamer sensor, resulting in an increasing electron transfer resistance of [Fe(CN)_6_]^3−/4−^.Figure 7B shows the variation of electron transfer resistance with CEA concentration. It can be seen from the figure that there is a good linear relationship between the electrochemical aptamer sensor and CEA when the CEA concentration is in the range of 0.2–15.0 ng/ml. The detection limit is 0.085 ng/ml (S/N = 3). The selectivity study was carried out to compare the sensor's selectivity performance of 10 ng/ml of CEA against DHEA, AFP, Leptin, AA, BSA and UA. The results demonstrated high selectivity with standard deviation less than 10%.

## Conclusion

Graphene ink was used to prepare SPE. a simple and fast unlabeled impedance-based electrochemical aptamer sensor for CEA detection was constructed by electrochemical deposition of AuNPs on the surface of SPE working electrode using cyclic voltammetry. CEA-A was immobilized on the electrode surface by the sulfhydryl self-assembly technique. After optimizations, the sensor can linear detection of CEA in the range of 0.2–15.0 ng/ml. The detection limit is 0.085 ng/ml (S/N = 3).

## Data Availability

The original contributions presented in the study are included in the article/supplementary material, further inquiries can be directed to the corresponding author.
